# Estimation of Knee Joint Forces in Sport Movements Using Wearable Sensors and Machine Learning

**DOI:** 10.3390/s19173690

**Published:** 2019-08-25

**Authors:** Bernd J. Stetter, Steffen Ringhof, Frieder C. Krafft, Stefan Sell, Thorsten Stein

**Affiliations:** 1 Institute of Sports and Sports Science, Karlsruhe Institute of Technology, 76131 Karlsruhe, Germany; 2 Department of Sport and Sport Science, University of Freiburg, 79117 Freiburg, Germany; 3 Joint Center Black Forest, Hospital Neuenbuerg, 75305 Neuenbuerg, Germany

**Keywords:** inertial sensors, artificial neural network, biomechanics, inverse dynamics

## Abstract

Knee joint forces (KJF) are biomechanical measures used to infer the load on knee joint structures. The purpose of this study is to develop an artificial neural network (ANN) that estimates KJF during sport movements, based on data obtained by wearable sensors. Thirteen participants were equipped with two inertial measurement units (IMUs) located on the right leg. Participants performed a variety of movements, including linear motions, changes of direction, and jumps. Biomechanical modelling was carried out to determine KJF. An ANN was trained to model the association between the IMU signals and the KJF time series. The ANN-predicted KJF yielded correlation coefficients that ranged from 0.60 to 0.94 (vertical KJF), 0.64 to 0.90 (anterior–posterior KJF) and 0.25 to 0.60 (medial–lateral KJF). The vertical KJF for moderate running showed the highest correlation (0.94 ± 0.33). The summed vertical KJF and peak vertical KJF differed between calculated and predicted KJF across all movements by an average of 5.7% ± 5.9% and 17.0% ± 13.6%, respectively. The vertical and anterior–posterior KJF values showed good agreement between ANN-predicted outcomes and reference KJF across most movements. This study supports the use of wearable sensors in combination with ANN for estimating joint reactions in sports applications.

## 1. Introduction

Knee pain and injury are common problems in both elite and recreational athletes in team and individual sports, and represent a large part of the costs of medical care [1]. Studies have highlighted that team sports that involve start–stop movements, rapid changes in direction, intense jumps and landings are prone to knee injuries [2,3]. Furthermore, epidemiological studies in team sports [4] and individual sports, such as running [5], found the knee to be one of the most frequently injured parts of the human body. The knee, as an important load-bearing joint in the body, undergoes huge stress during activities, due to the multidirectional forces exerted on the joint [6,7,8,9]. Therefore, forces transmitted by the knee are of great significance, as they provide a resource to estimate the internal loading of the anatomical structures (e.g., bones) [10,11].

A common way of assessing the load on internal anatomical structures is through the use of biomechanical modelling. Inverse dynamics can be calculated by means of three-dimensional (3D) motion capture and force plate data [12]. Inverse dynamics studies have been carried out to determine knee kinetics during various movements, such as walking [13,14], running [14,15], cutting [16,17], and jumping [18,19]. It must be noted that two different types of knee forces can be calculated by means of biomechanical modelling. First are net joint forces (also termed as joint intersegmental forces or joint reaction forces), calculated using the traditional Newton–Euler inverse dynamics method [12,20]; second are joint contact forces, representing the sum of the net joint forces and the compressive joint forces [10,20,21]. The compressive joint forces are mainly caused by muscle forces, and can be obtained via musculoskeletal modelling [10,20,21]. Knee joint contact forces have additionally been measured in vitro by means of an instrumented implant [8,9]. Therein, it was shown that knee joint contact forces are closely related to the activity [8,9]. High-impact activities, such as tennis, generate peak tibial forces of up to four times the body weight [8]. Net joint forces underestimate the actual internal load, but their determination require less complex modelling [10,20]. However, neither the biomechanical modelling nor direct force measurement can be readily added to an athletes’ natural sports environment.

As a consequence, alternative technologies, such as wearable inertial measurement units (IMUs), have experienced tremendous advances within the last two decades [22,23]. The integration of such sensors into sports equipment (e.g., shoes) or attachment to an athlete has allowed the assessment of temporal, kinematic, and dynamic parameters [23]. The recent review by Camomilla et al. [23] highlighted the potential of wearable inertial sensors for sports performance evaluation. However, performance indicators are not necessarily appropriate to characterize the loads on specific body structures, especially joints. The estimation of biomechanical variables has not yet been fully established, primarily due to the difficulty in assessing external forces [23].

Recently, estimating the ground reaction force (GRF) by means of wearable sensors has gained more attention [24,25]. The majority of applied methods require modelling of the musculoskeletal system to a certain extent, which requires subject-specific data (e.g., mass, dimensions, and center of mass of the body segments), which inevitably introduce inaccuracies and uncertainty [24,26,27]. As a consequence, several studies have explored modern machine learning techniques to simplify modelling and data acquisition strategies [27,28,29]. The recent study by Wouda et al. [28] presented an artificial neural network (ANN) approach to estimate vertical GRF during running, based on vertical accelerations and lower limb joint angles. The estimated GRF profiles of the non-personalized ANN showed a high correlation (>0.90) with the actual force time series. Guo et al. [27] used directly-measured acceleration signals without providing joint kinematics, as well as a slightly different model (nonlinear, autoregressive moving average model with exogenous inputs) to estimate vertical GRF during walking. In this study, a minimum model prediction error of 3.8% was shown when comparing the predicted vertical GRF time series to data measured directly from pressure insoles. Although the studies described above have estimated forces during locomotion, no study has yet performed a direct estimation of knee joint forces; these are of paramount importance, as the GRF is not necessarily an accurate predictor of knee joint loading, due to modulations within the kinetic chain of the lower extremity [13,15,30].

In summary, having a field-based method to quantify and monitor knee joint forces in the field is of substantial importance from two viewpoints: (1) studying the relationship between force measures and injury helps to establish effective injury prevention strategies; and (2) to monitor an athlete’s workload is important in setting up effective training programs, which provide an adequate training stimulus while minimizing the risk of non-functional overreaching (e.g., pain). Providing this feedback to athletes, coaches, and physicians is highly relevant, especially during rehabilitation after an injury.

Therefore, the purpose of this study was to develop an ANN that estimates net knee joint forces during sport movements, based on data obtained by wearable sensors. The findings of this study could help to overcome current restrictions in the mobile assessment of knee joint forces and open new possibilities for in-field diagnosis, which could help to provide better injury prevention strategies in the future.

## 2. Materials and Methods

### 2.1. Participants

A total of 13 healthy male sport students (age: 26.1 ± 2.9 years; height: 178.7 ± 5.5 cm; body mass: 78.4 ± 5.9 kg) voluntarily participated in this study. None reported recent injuries. The study was approved by the ethics committees of the Karlsruhe Institute of Technology. All participants were informed of the experimental procedures and gave informed written consent prior to the test.

### 2.2. Measurement Protocol

All participants came once to the institutional motion analysis laboratory. After signing the study documents, anthropometric measurements were taken, and the data collection equipment was attached. After warming up by running on a treadmill for 5 min at a self-selected speed, participants were instructed to perform a variety of sport-specific movements, including moderate running; fast running; running 90° clockwise turns; running 90° counterclockwise turns; sprint start; full-stop after sprinting; left-sided cutting maneuver; right-sided cutting maneuver; side shuffle cut; straight ahead walking; walking 90° clockwise turns; walking 90° counterclockwise turns; one-leg horizontal jumps (submaximal; distance = 50% body height); and maximal, two-leg, vertical counter movement jumps. For a detailed description of the cutting maneuver (called a “v-cut”) and side shuffle cut, see Neptune et al. [31]. The 90° turns were carried out following Krafft et al. [32].

### 2.3. Measurement Setup

**Figure 1:**
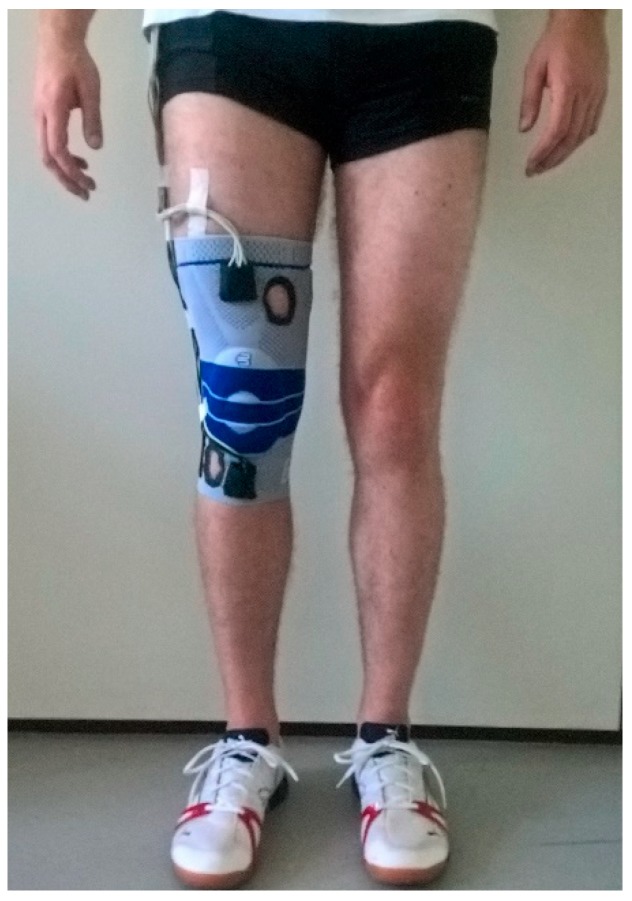
Placement of inertial measurement units (IMUs) used in the study. The IMUs were positioned in the two black patch pockets at the upper and lower frontal end of the sleeve.

Full-body kinematics were recorded with a marker-based motion capture system (11 MX-13 cameras, 200 Hz, Vicon, Oxford, UK). A total of 42 spherical reflective markers were placed on the participants’ skin using the ALASKA Dynamicus protocol (ALASKA, INSYS GmbH, Germany). 3D GRF data were collected simultaneously from two plates (1000 Hz; AMTI Inc., Watertown, MA, USA) embedded in the floor and centered in the capture volume. Two identical, custom-built, six-degrees-of-freedom IMUs (1500 Hz, ±8 g accelerometer, ±2000°/s gyroscope) were attached to each participant’s right leg via a knee sleeve, in order to capture IMU signals related to knee kinematics and dynamics. The IMUs were positioned at the upper and lower frontal end of the sleeve (Figure 1), and connected to a data acquisition unit. The data collecting systems were synchronized during post-processing by an analog signal, induced by the 3D motion capture system each time data acquisition was initiated.

### 2.4. Data Processing and Biomechanical Modelling

The 3D trajectories of the markers were reconstructed using Vicon Nexus V1.8.5. After 15 Hz low-pass filtering (Butterworth fourth-order filter) of the 3D marker coordinates and GRF data [33], net knee joint forces (KJF) ($F_{v}$ = vertical, $F_{ap}$ = anterior–posterior, and $F_{ml}$ = medial–lateral component) were determined via inverse dynamic modelling, using the full-body Dynamicus 9 model [34,35]. Each participant was individually modeled as a linked-segment model based on standardized anthropometric measures [36]. By means of the recorded full-body kinematics and external forces, inertial net forces were calculated [12]. A 20 N threshold of the vertical GRF was used to extract the stance phase for each locomotion movement [37]. Two separate stance phases were extracted for the jumps. The first represented take-off, starting at the time point when vertical GRF undercut the body weight, and ending when vertical GRF was zero (beginning of the flight phase). The second stance phase represented the landing, starting when vertical GRF was greater than zero (end of the flight phase), and ending when vertical GRF equaled body weight. As a consequence, each of the two jump forms consisted of two conditions, whereas the other twelve movements consisted of one condition. Ten trials were excluded from the inverse dynamic calculation, due to measurement errors of the 3D motion capture system, resulting in a total of 198 trials (13 participants’ × 16 conditions – 10 invalid trials).

The IMU signals were also filtered (Butterworth fourth-order filter; cut-off frequency of 15 Hz) and each trial was cropped to contain data for the same phase as the KJF. Subsequently, the KJF time series and IMU signals were organized to represent 0%–100% of the stance phase. Finally, an IMU signal matrix and a KJF matrix were created by vertically concatenating the IMU signals and KJF time series, respectively, of all the trials. Both matrices contained 19,800 rows (198 trials × 100 time points), with 12 columns for the IMU signal matrix (six acceleration signals + six angular velocity signals) and three columns for the KJF matrix (three spatial dimensions).

### 2.5. Neural Network Modelling

The ANN developed for this study maps the IMU signals of all movements to the KJF time series of all movements, and was set up with the Neural Network Toolbox in MATLAB R2018b (The MathWorks, United States). The IMU signal matrix served as the input and the KJF matrix served as the target (output). Thus, the ANN had 12 variables (i.e., nodes) in its input layer and three variables in its output layer. The ANN had two hidden layers, one with 250 and one with 100 neurons, which were connected to the input and output nodes [28]. The ANN was trained with a Levenberg–Marquardt, back-propagated error correction, and a random division of 70/15/15 was used for the respective training/validation/testing. Hyperbolic tangent sigmoid activation functions were defined between the hidden layers. The network was trained for 1000 iterations, and training was stopped if the gradient did not decrease for six consecutive iterations, or if the gradient was smaller than 1 × 10^−6^. Evaluation of the ANN was done using a leave-one-subject-out cross-validation, in order to assess the performance of a non-personalized model. The cross-validation involved training the ANN with all trials from 12 participants (i.e., the training set), and then testing with the trials from the remaining participant (i.e., the test set).

### 2.6. Statistical Analysis

For each movement, the similarity between the ANN-predicted KJF time series ($F_{v}^{*}$, $F_{ap}^{*}$ and $F_{ml}^{*}$) and the calculated inverse dynamics ($F_{v}$, $F_{ap}$ and $F_{ml}$) was assessed using Pearson’s correlation coefficient (*r*) and relative root-mean-squared error (rRMSE) [38]. The averages and standard deviations from the 13 cross-validation subsets were calculated for *r* and the rRMSE. A Fishers *z*-transformation of *r* was performed to calculate the mean correlation coefficient. Mean values were expressed as *r* by reversing the transformation. Additionally, classical discrete biomechanical metrics of knee loading were evaluated by means of peak $F_{v}$ and summed $F_{v}$ over the stance phase. Percent differences (*%Diff*) between ANN-predicted peak $F_{v}$, inverse-dynamic calculated peak $F_{v}$, and summed $F_{v}$ were used to provide a pragmatic interpretation.

## 3. Results

**Figure 2:**
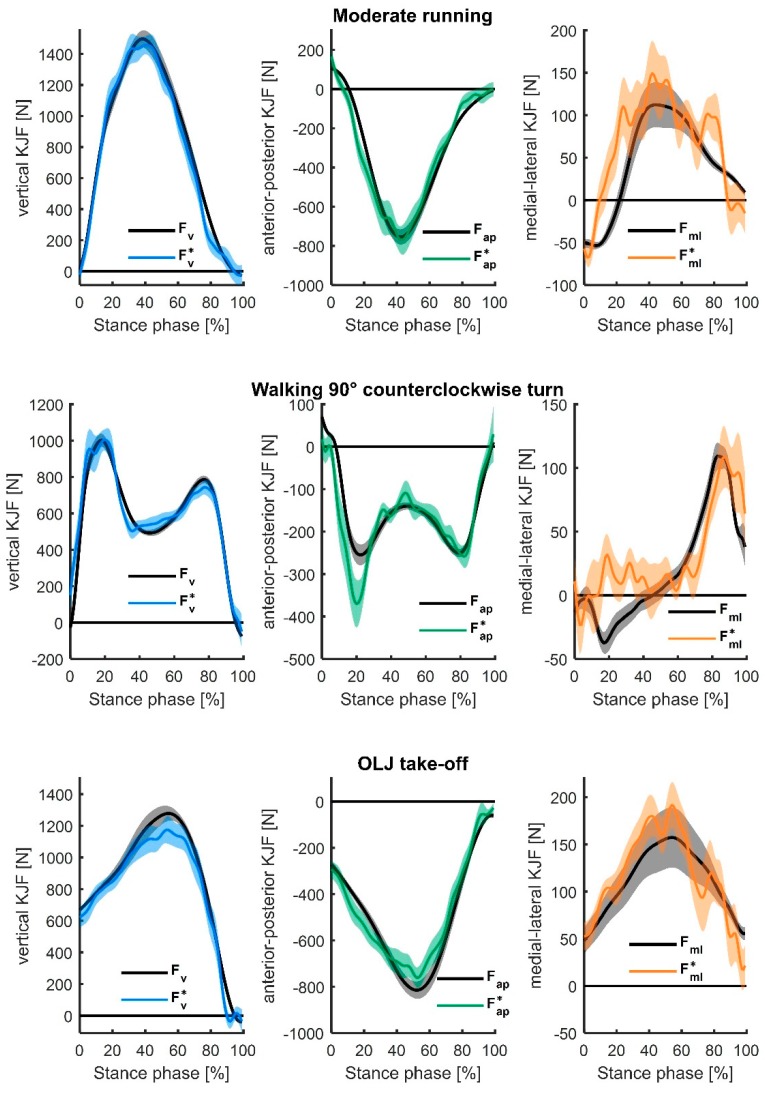
Mean (and standard error) of the estimated three-dimensional (3D) knee joint forces (KJF) ($F_{v}^{*},\;F_{ap}^{*}$, and $F_{ml}^{*}$) for moderate running (top), walking a 90° counterclockwise turn (middle), and one-leg horizontal jump (OLJ) take-off are presented (normalized to the stance phase), compared to their respective reference values (inverse dynamics-calculated knee joint forces *F_v_*, *F_ap_*, and *F_ml_*).

Table 1 shows an overview of the estimated accuracy for all movements. The ANN-predicted KJF yielded *r* values that ranged from 0.60 to 0.94 ($F_{v}^{*}$ vs. $F_{v}$), 0.64 to 0.90 ($F_{ap}^{*}\;$ vs. $F_{ap}$) and 0.25 to 0.60 ($F_{ml}^{*}$ vs. $F_{ml}$) for the different movements. $F_{v}^{*}$ for moderate running showed the highest correlation with $F_{v}$ (0.94 ± 0.33). The rRMSE between $F_{v}^{*}$ and $F_{v}$, $F_{ap}^{*}$ and $F_{ap}$, and $F_{ml}^{*}$ and $F_{ml}$ ranged between 14.2% and 25.9%, 17.4% and 27.1%, and 27.7% and 45.9%, respectively. The estimation of $F_{v}$ for moderate running and walking yielded the lowest rRMSE (14.2% each). The time series of estimated KJF are shown in Figure 2 for three representative movements (moderate running, walking a 90° counterclockwise turn, and a one-leg horizontal jump take-off). The KJF time series of all movements are provided as Supplementary Materials (Data File S1).

Results of the discrete outcomes (peak $F_{v}$ and summed $F_{v}$) are presented in Table 2. The mean peak $F_{v}$ difference between ANN-predicted and the reference values across all movements was 17.0% ± 13.6%. The smallest *%Diff* values were seen for side shuffle cut (2.6% ± 19.3%). Differences between the ANN-predicted and the reference values were smaller for the summed $F_{v}$ (mean differences across all movements = 5.7% ± 5.9%) compared to the peak $F_{v}$ (mean differences across all movements = 17.0% ± 13.6%). Of the 16 movements, 13 had a *%Diff* for summed $F_{v}$ smaller than 6.8%. Two-leg jump take-off and landing yielded substantial differences for both metrics (*%Diff* peak *F_v_* ≥ 22.9%; *%Diff* summed *F_v_* ≥ 16.1%).

## 4. Discussion

This study investigated the feasibility of an ANN approach to estimate KJF during sport-specific movements based on data from two IMUs. Mobile assessment of KJF allows measurement of biomechanics outside the laboratory. The accuracy of ANN estimation of various common sport-specific movements was compared to standard inverse dynamic-calculated KJFs.

The results indicated that the estimation accuracy of the ANN varied between movements, but that accuracy was good for most movements. With respect to the three different force components, vertical KJF showed the highest agreement between the ANN-predicted outcomes and the inverse dynamics-calculated data, followed by the anterior–posterior KJF, and finally the medial–ateral. For 13 of the 16 movements, discrete biomechanical measures showed a *%Diff* for summed $F_{v}$ of less than 6.8%; and 12 of the 16 movements showed a *%Diff* for peak $F_{v}$ of less than 19.3%.

### 4.1. Comparison of Different Movements

In general, good agreement (*r* ≥ 0.81 and rRMSE ≤ 20.3%) was found for $F_{v}$ of the majority (11 out of 16) of the analyzed movements. Ten of the 16 movements showed comparable estimation accuracies (*r* ≥ 0.80 and rRMSE ≤ 22.9%) for $F_{ap}$. However, there was a pronounced drop (*r* ≤ 0.60 and rRMSE ≥ 27.7%) in estimation accuracy for $F_{ml}$.

When comparing the estimation accuracy for $F_{v}$ across the different movements, moderate running had the highest predictive power. Alterations of the running movement, such as running turns and cutting maneuvers, as well as walking forms, showed slight reductions in the estimation accuracy. A potential reason for the higher predictive power of moderate running is the repeatable characteristic of the movement, while other movements are performed with a higher rate of variation [39]. Similar changes in estimation accuracy were shown by Fluit et al. [40] when they evaluated a prediction model for GRFs and moments during various activities of daily living, by means of 3D full-body motion. The limited estimation accuracy for sprint starts, full-stops, and side shuffle cuts may be explained by higher variations in the execution of such movements. Reduced estimation accuracy in continuous outcomes does not necessarily mean an inaccurate estimation of discrete variables, as seen for full stops. However, it must be noted that both variables show a high standard deviation, which indicates a wide dispersion across participants.

Across all movements, differences for summed $F_{v}$ were lower than for peak $F_{v}$. Therefore, estimated peak $F_{v}\;$ should be treated with caution. The ANN often overestimated the peak $F_{v}$, but slightly underestimated the summed $F_{v}$ for the majority of the movements. A study by Charry et al. [41], which investigated the predictive ability of tibial accelerations to estimate peak vertical GRF in running, showed lower deviations (rRMSE ≈ 6%) for comparable discrete variables. However, their method was only applied to training and testing on individuals. Distinct differences (*r* for take-off: 0.92 vs. 0.60; *r* for landing: 0.84 vs. 0.61) in estimation accuracy were seen between one-leg jumps and two-leg jumps, respectively. Additionally, a high *%Diff* was seen between ANN-predicted and inverse dynamics-calculated peak $F_{v}$ and summed $F_{v}$ for two-leg jumps. One reason for the reduced estimation accuracy for two-leg jumps may be the bipedal characteristic of the movement. Potential inaccuracies in KJF estimations are caused by the distribution of the total external load on both legs. Combining knee joint force estimations with an activity recognition approach could help to overcome such limitations by selecting individual prediction models for movement categories.

### 4.2. Comparison with Related Methods

Overall, machine learning-based approaches do not need an a priori knowledge of the model or require modelling of the musculoskeletal system, since they build up their model as they go using training data [24]. It should be noted that ground truth reference data, such as the calculated KJFs by means of biomechanical modelling, are necessary for the model development process. Such methods run on the hypothesis that a relationship exists between the sensor signals measured somewhere on the body and the biomechanical target variable (e.g., KJF) [24]. This is supported by the relationship between acceleration and force, according to Newton’s second law of motion, as well as by the relationship between the measured quantities and the segment’s motion [26,28]. There are many studies highlighting the usability of ANN to estimate GRF or joint moments by means of kinematic data obtained from an optical motion analysis system [24,42,43,44]. In contrast to a machine learning-based approach, kinematics and kinetics of the lower limb joints can be estimated by means of posture information obtained from wearable sensors and an analytical model [45]. Such approaches typically require the modelling of the biomechanical system (e.g., trunk, thigh, shank, and foot) to a certain extent. For the most part, complex modelling is necessary to obtain reasonable results, as discrepancies in subject-specific parameters, such as masses, dimensions, etc., inevitably introduce inaccuracies [24].

One of the first studies combining wearable sensors and ANN was done by Leoprace et al. [29]. Their findings revealed the lowest *r* in the medial–lateral component when they estimated 3D GRF with an ANN during walking. In the present study, the medial–lateral KJF were also found to have the lowest *r* value compared to the other two components across all investigated movements. Previous studies estimating the GRF highlighted similar findings and suggested that this was due to the small magnitude of the lateral measurements, which causes a larger impact of small errors on final estimates [26,40,44]. Karatsidis et al. [26] compared the GRF estimation accuracy of a full-body inertial motion capture and optical motion capture system. Their results showed slightly higher *r* values (ranging from to 0.82 to 0.99 and 0.76 to 0.99 for the inertial and optical motion capture systems, respectively) and lower rRMSE values (ranging from around 5% to 15% for both systems) for walking than the present study.

Wouda et al. [28] used a similar machine-learning approach to the one we used for estimating vertical GRF and sagittal knee kinematics during running. The estimated vertical GRF profiles of their non-personalized ANN showed a slightly higher agreement (*r* > 0.94) with the actual force time series for five of their eight participants. One reason for the slightly lower accuracy in our study may be the less specific model with respect to single movements. Overall, a more generic model for multiple movements decreases the performance for some movements (as described above), but provides the advantage that not every movement must be modeled. From a practical point of view, this ultimately enlarges the use. However, it remains unclear if the level of accuracy would be high enough for applications of interest, such as tracking fatigue-related changes, which may be related to increased chance of injury [46]. This is especially due to the fact that research in this area is limited, and much of what we know about monitoring comes from personal experience [46]. Future research in applied settings would be indispensable to observe and analyze biomechanical risk factors over a defined exposure time, with the ability to influence injury prevention models [47].

### 4.3. Limitations

Attention was paid to the fixation of the IMUs to limit their oscillations and any misalignment; however, we cannot fully ensure that the fixation technique excluded this source of error, due to the explosive characteristic of some tasks. To control for this issue, the exact fit of the sleeve was checked periodically and replaced when necessary. Additionally, the IMUs measure acceleration and angular velocity on the body surface, and relative movements may occur with respect to the bone [48]. Such movements may negatively affect the estimation of the KJFs, especially for movements that are highly dynamic.

As the estimation accuracy of the proposed approach depends on the neural network architecture, this is a potential limitation of the study. Our ANN was built in accordance with previous work [28], and is capable of mapping non-linearity between input and output; however, we cannot exclude that other model specifications would result in an improved outcome. In addition, a relatively small sample size was used to build the ANN. This represents a limitation of the study, as the robustness of the relationship between the input and output variables of the ANN depends on the amount of training data [24]. The ANN was trained with data from all tested movements. As a consequence, it remains unclear to which extent an ANN built with a subset of movements could estimate KJFs of movements that were not included in the training of the model. It is worth noting that the net KJF used to build the ANN represent only a part of the internal loading of the anatomical structures. Muscles forces, which contribute to the total force transmitted by the joint, were not incorporated in the biomechanical modelling [10,21].

In the current approach, direct acceleration and angular velocity measures were input to the ANN. The amplitudes of such signals are sensitive to the placement and fixation technique, as well as participant anthropometrics and soft tissue characteristics [49]. In order to keep potential artefacts low, this study involved only young and healthy male sport students. Further research is necessary to better assess the effects of inter-participant variabilities on input signals for the model building, as well as to translate the results of this study to other age and sex groups or athletes in rehabilitation. Body weight normalization of the KJF time series could help to compensate for variations across individuals.

## 5. Conclusions

The results of this study show that a machine-learning approach can be very useful to estimate KJF for various movements based on data obtained by two wearable sensors. Specifically, the vertical and anterior–posterior KJF showed good agreement between the ANN-predicted outcomes and inverse dynamic-calculated forces for a variety of movements. However, caution is required for tasks with lower estimation accuracy (e.g., two-leg jumps). It could be helpful to develop individual prediction models for movement categories, such as bilateral tasks, in order to strengthen the overall estimation accuracy. Additionally, a comparison of ANN with different configurations and inputs could help to improve the estimation accuracy, as well as perform a sensor-to-segment calibration for aligning wearable sensors with human body segments. The scaling of input signals (e.g., acceleration signals to body mass) or the normalization of the KJF time series to body weight could help to compensate for inter-individual differences. Future research could focus on the combination of the presented approach with musculoskeletal modelling or with direct force measurements, using an instrumented knee prosthesis. Providing the best means of reference data for the ANN modelling could help to assess the internal loadings on the knee joint structures more precisely. Looking ahead, this study supports the use of wearable sensors in combination with machine-learning techniques for estimating joint reactions in sports applications. Ultimately, this has high practical implications, as new possibilities for in-field diagnosis can help to provide better injury prevention strategies in the future.

## Figures and Tables

**Table 1:** Accuracy (*r*: Pearson’s correlation coefficient; rRMSE: relative root-mean-squared error) of the predicted continuous knee joint force outcomes (vertical (F*_v_), anterior–posterior (F*_ap_), and medial–lateral (F*_ml_)). Values are presented as mean (and standard deviation).

Movement Task	Component					
	F*_v_		F*_ap_		F*_ml_	
	*r*	rRMSE [%]	*r*	rRMSE [%]	*r*	rRMSE [%]
Moderate running	0.94 (0.33)	14.2 (4.0)	0.90 (0.30)	18.9 (5.5)	0.43 (0.26)	41.7 (11.5)
Fast running	0.89 (0.43)	20.3 (5.8)	0.88 (0.44)	22.9 (9.5)	0.42 (0.41)	43.4 (12.0)
Running 90° clockwise turn	0.89 (0.40)	17.2 (4)	0.82 (0.36)	21.0 (6.5)	0.38 (0.35)	36.7 (18.4)
Running 90° counter-clockwise turn	0.87 (0.35)	17.5 (5.3)	0.88 (0.43)	19.5 (8.1)	0.37 (0.42)	37.2 (11.5)
Sprint start	0.73 (0.45)	25.9 (8.8)	0.76 (0.40)	25.8 (9.3)	0.31 (0.29)	43.3 (10.0)
Full-stop	0.78 (0.45)	24.7 (7.2)	0.80 (0.34)	21.8 (7.5)	0.45 (0.29)	37.7 (9.0)
Left-sided cutting maneuver	0.86 (0.44)	19.4 (6.6)	0.86 (0.41)	22.0 (7.3)	0.30 (0.42)	44.8 (13.0)
Right-sided cutting maneuver	0.86 (0.39)	19.0 (5.4)	0.84 (0.35)	21.5 (5.2)	0.25 (0.39)	45.7 (9.0)
Side shuffle cut	0.79 (0.47)	20.4 (6.6)	0.81 (0.43)	19.8 (6.0)	0.35 (0.45)	36.5 (9.3)
Walking	0.87 (0.32)	14.2 (4.3)	0.71 (0.39)	20.8 (5.6)	0.60 (0.31)	27.7 (5.7)
Walking 90° clockwise turn	0.81 (0.27)	16.9 (4.5)	0.65 (0.31)	23.0 (6.2)	0.31 (0.20)	34.1 (8.1)
Walking 90° counter-clockwise turn	0.83 (0.29)	15.3 (4.0)	0.64 (0.30)	22.7 (5.8)	0.48 (0.34)	29.1 (6.0)
One-leg jump take-off	0.92 (0.39)	15.4 (6.6)	0.89 (0.25)	17.4 (5.5)	0.31 (0.46)	45.9 (19.7)
One-leg jump landing	0.84 (0.43)	16.7 (7.2)	0.77 (0.53)	25.1 (9.4)	0.42 (0.38)	38.9 (14.4)
Two-leg jump take-off	0.60 (0.36)	23.0 (8.6)	0.82 (0.40)	20.5 (7.4)	0.51 (0.23)	27.8 (2.9)
Two-leg jump landing	0.61 (0.34)	25.9 (6.2)	0.65 (0.36)	27.1 (5.5)	0.54 (0.32)	37.6 (6.8)
Mean	0.82 (0.10)	19.1 (4.0)	0.79 (0.09)	21.8 (2.6)	0.40 (0.10)	38.0 (6.1)

**Table 2:** Absolute percent differences (*%Diff*) between ANN-predicted peak, inverse dynamic-calculated peak, and summed vertical knee joint force (*Fv*). The superscript minus indicates an underestimation of the ANN.

Movement Task	Discrete Biomechanical Metrics	
	Peak *F_v_*	Summed *F_v_*
	*%Diff*	*%Diff*
Moderate running	10.0 (12.8)	3.0 (11.0)^‾^
Fast running	16.1 (34.2)	2.8 (15.5)^‾^
Running 90° clockwise turn	17.4 (36.3)	6.8 (15.2)^‾^
Running 90° counter-clockwise turn	19.3 (28.0)	2.3 (9.6)^‾^
Sprint start	24.9 (26.7)	1.5 (31.0)^‾^
Full-stop	3.3 (23.3)	2.6 (32.0)^‾^
Left-sided cutting maneuver	21.0 (25.6)	0.8 (15.8)
Right-sided cutting maneuver	17.2 (20.2)	1.9 (16.4)
Side shuffle cut	2.6 (19.3)^‾^	15.0 (7.3)^‾^
Walking	13.8 (16.2)	0.9 (9.3)
Walking 90° clockwise turn	8.7 (12.6)	2.1 (12.7)
Walking 90° counter-clockwise turn	19.5 (24.5)	2.6 (7.5)
One-leg jump take-off	8.0 (18.7)	6.5 (17.0)^‾^
One-leg jump landing	6.4 (12.6)^‾^	6.1 (10.5)^‾^
Two-leg jump take-off	60.8 (59.8)	16.1 (31.2)
Two-leg jump landing	22.9 (34.7)	19.5 (30.0)
Mean	17.0 (13.6)	5.7 (5.9)

## Supplementary Materials

The following are available online at https://www.mdpi.com/1424-8220/19/17/3690/s1, Data File S1: Knee joint forces (KJF) time series of all movements.
