# Supplementary material for: Estimation of Knee Joint Forces in Sport Movements Using Wearable Sensors and Machine Learning
Source: Sensors (Basel). 2019 Aug 25;19(17):3690. doi: 10.3390/s19173690 (PMC6749227; doi:10.3390/s19173690)
Supplement: Supplementary file 1 [file sensors-19-03690-s001.pdf]

Supplementary Figures: Knee joint forces (KJF) time series of all movements

r: Pearson's correlation coefficient; rRMSE: relative root-mean-squared error; OLJ: one-leg jump; TLJ: two-leg jump; mean (standard deviation)

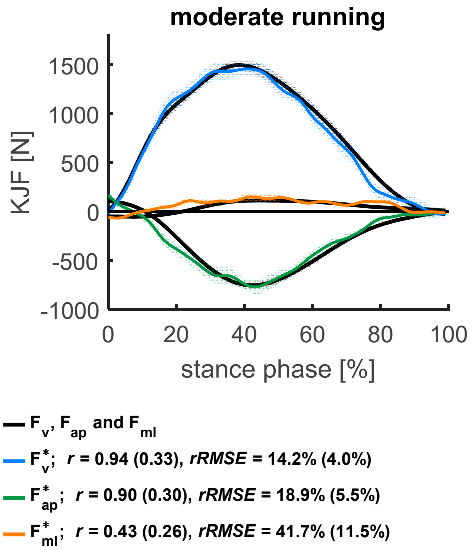

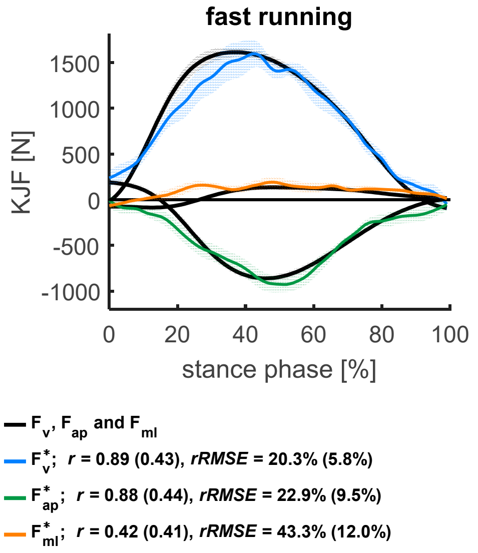

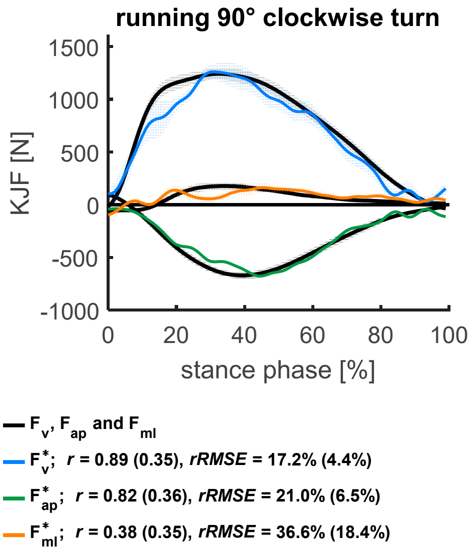

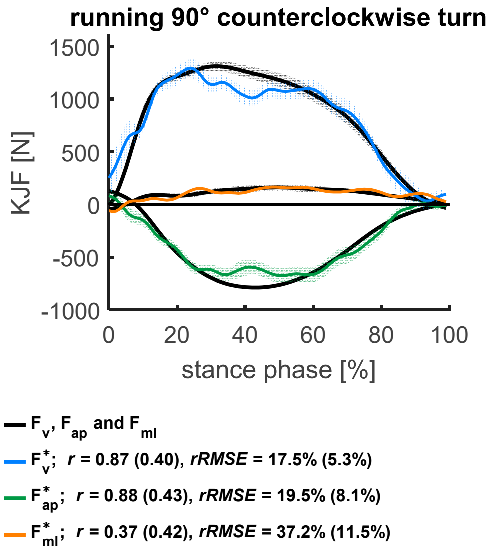

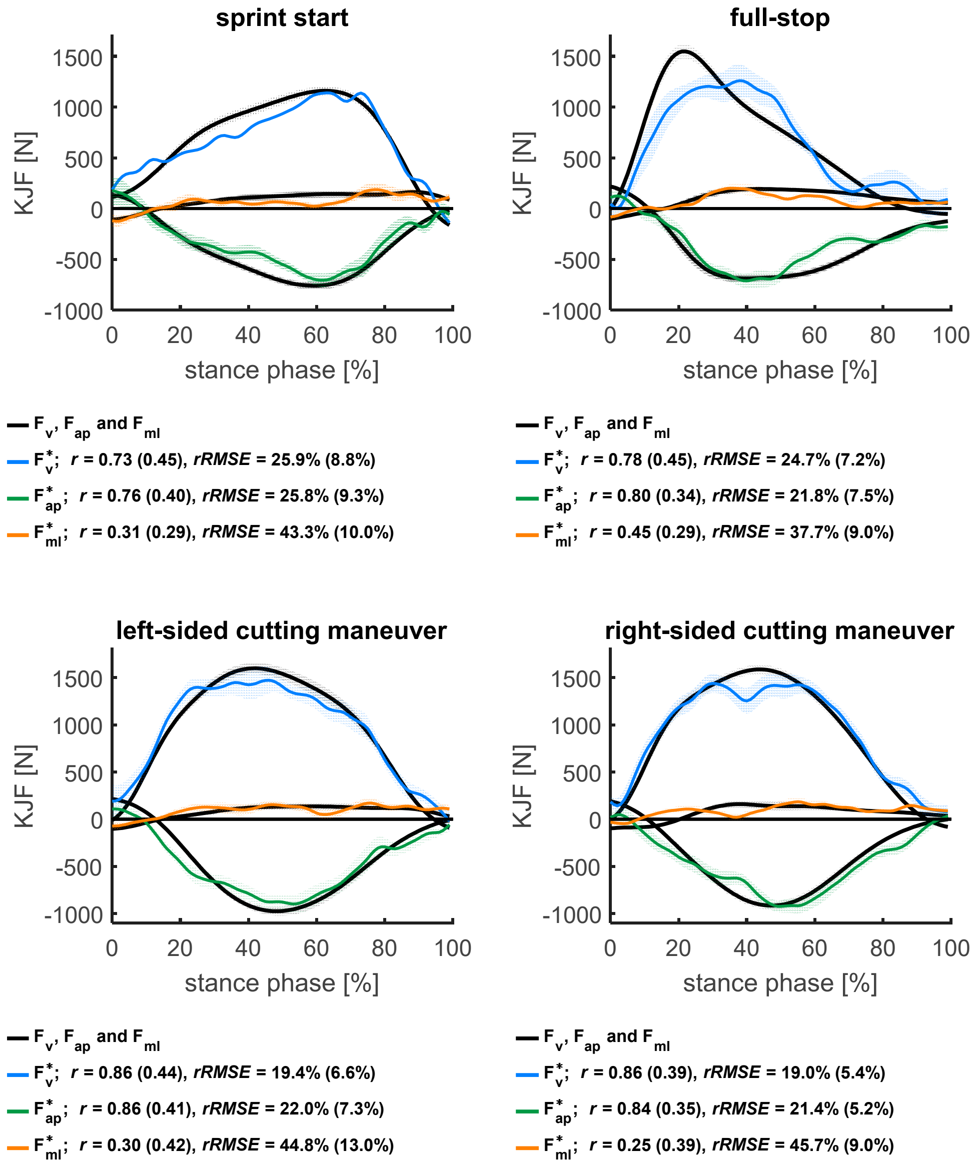

sprint start
full-stop
left-sided cutting maneuver
right-sided cutting maneuver
KJF [N]
stance phase [%]
1500
1000
500
0
-500
-1000
0
20
40
60
80
100
Fv, Fap and Fml
F*v; r = 0.73 (0.45), rRMSE = 25.9% (8.8%)
F*ap; r = 0.76 (0.40), rRMSE = 25.8% (9.3%)
F*ml; r = 0.31 (0.29), rRMSE = 43.3% (10.0%)
Fv, Fap and Fml
F*v; r = 0.78 (0.45), rRMSE = 24.7% (7.2%)
F*ap; r = 0.80 (0.34), rRMSE = 21.8% (7.5%)
F*ml; r = 0.45 (0.29), rRMSE = 37.7% (9.0%)
Fv, Fap and Fml
F*v; r = 0.86 (0.44), rRMSE = 19.4% (6.6%)
F*ap; r = 0.86 (0.41), rRMSE = 22.0% (7.3%)
F*ml; r = 0.30 (0.42), rRMSE = 44.8% (13.0%)
Fv, Fap and Fml
F*v; r = 0.86 (0.39), rRMSE = 19.0% (5.4%)
F*ap; r = 0.84 (0.35), rRMSE = 21.4% (5.2%)
F*ml; r = 0.25 (0.39), rRMSE = 45.7% (9.0%)

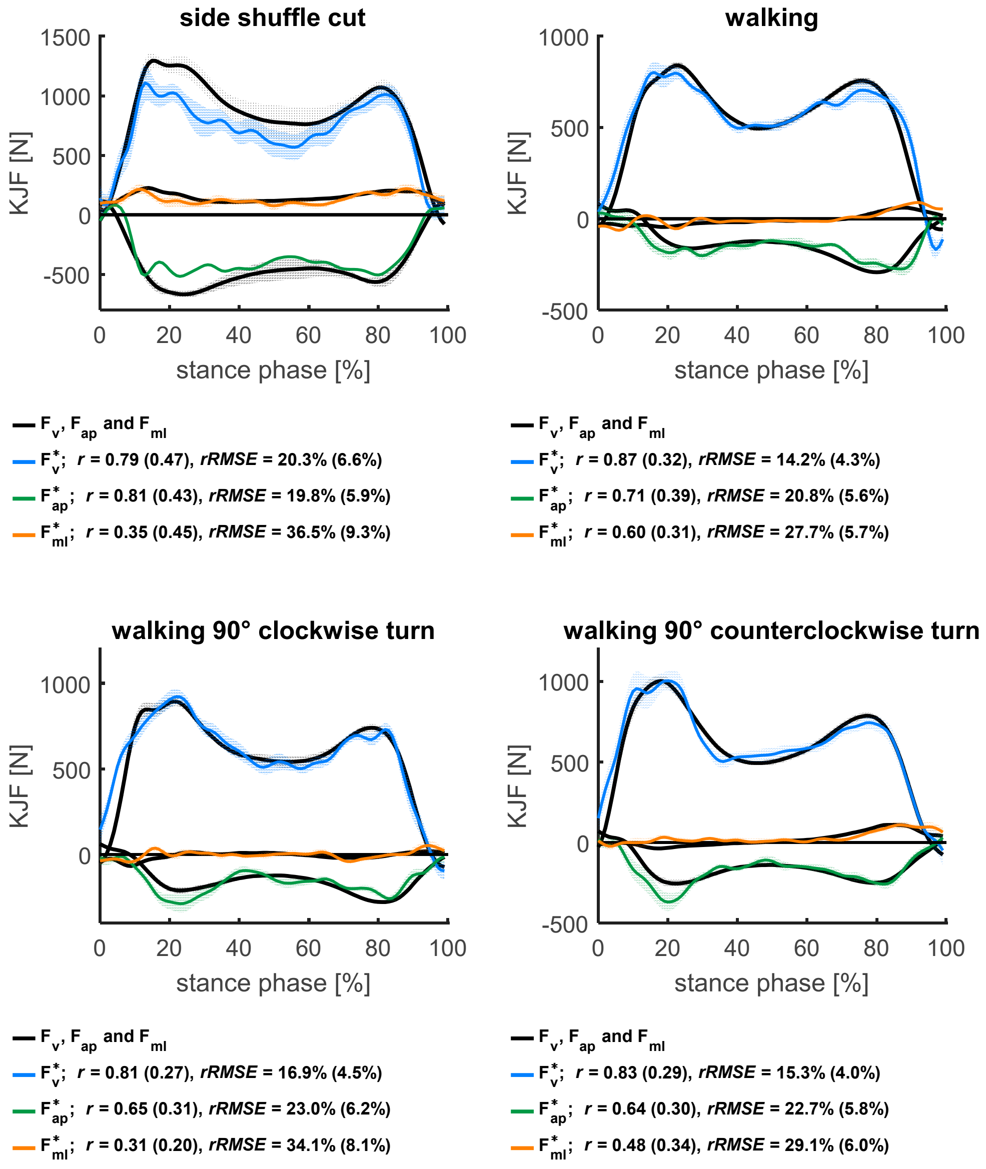
side shuffle cut
KJF [N]
1500
1000
500
0
-500
0
20
40
60
80
100
stance phase [%]
F_v, F_{ap} and F_{ml}
F^*_v; *r* = 0.79 (0.47), *rRMSE* = 20.3% (6.6%)
F^*_{ap}; *r* = 0.81 (0.43), *rRMSE* = 19.8% (5.9%)
F^*_{ml}; *r* = 0.35 (0.45), *rRMSE* = 36.5% (9.3%)
walking
KJF [N]
1000
500
0
-500
0
20
40
60
80
100
stance phase [%]
F_v, F_{ap} and F_{ml}
F^*_v; *r* = 0.87 (0.32), *rRMSE* = 14.2% (4.3%)
F^*_{ap}; *r* = 0.71 (0.39), *rRMSE* = 20.8% (5.6%)
F^*_{ml}; *r* = 0.60 (0.31), *rRMSE* = 27.7% (5.7%)
walking 90° clockwise turn
KJF [N]
1000
500
0
0
20
40
60
80
100
stance phase [%]
F_v, F_{ap} and F_{ml}
F^*_v; *r* = 0.81 (0.27), *rRMSE* = 16.9% (4.5%)
F^*_{ap}; *r* = 0.65 (0.31), *rRMSE* = 23.0% (6.2%)
F^*_{ml}; *r* = 0.31 (0.20), *rRMSE* = 34.1% (8.1%)
walking 90° counterclockwise turn
KJF [N]
1000
500
0
-500
0
20
40
60
80
100
stance phase [%]
F_v, F_{ap} and F_{ml}
F^*_v; *r* = 0.83 (0.29), *rRMSE* = 15.3% (4.0%)
F^*_{ap}; *r* = 0.64 (0.30), *rRMSE* = 22.7% (5.8%)
F^*_{ml}; *r* = 0.48 (0.34), *rRMSE* = 29.1% (6.0%)

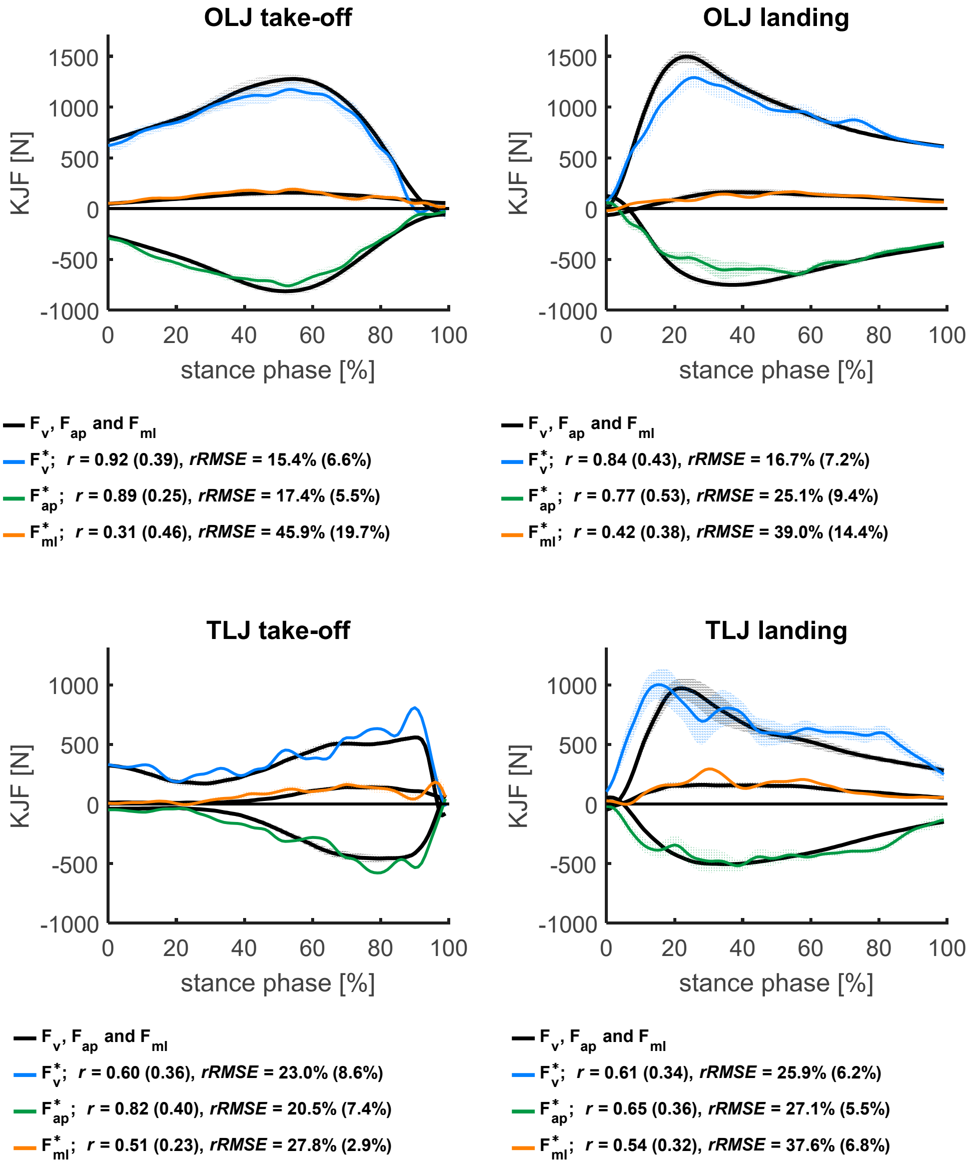

OLJ take-off
OLJ landing
TLJ take-off
TLJ landing
KJF [N]
stance phase [%]
1500
1000
500
0
-500
-1000
0
20
40
60
80
100
F v, F ap and F ml
F*v; r = 0.92 (0.39), rRMSE = 15.4% (6.6%)
F*ap; r = 0.89 (0.25), rRMSE = 17.4% (5.5%)
F*ml; r = 0.31 (0.46), rRMSE = 45.9% (19.7%)
F v, F ap and F ml
F*v; r = 0.84 (0.43), rRMSE = 16.7% (7.2%)
F*ap; r = 0.77 (0.53), rRMSE = 25.1% (9.4%)
F*ml; r = 0.42 (0.38), rRMSE = 39.0% (14.4%)
F v, F ap and F ml
F*v; r = 0.60 (0.36), rRMSE = 23.0% (8.6%)
F*ap; r = 0.82 (0.40), rRMSE = 20.5% (7.4%)
F*ml; r = 0.51 (0.23), rRMSE = 27.8% (2.9%)
F v, F ap and F ml
F*v; r = 0.61 (0.34), rRMSE = 25.9% (6.2%)
F*ap; r = 0.65 (0.36), rRMSE = 27.1% (5.5%)
F*ml; r = 0.54 (0.32), rRMSE = 37.6% (6.8%)
